# Declining Transmission and Immunity to Malaria and Emerging Artemisinin Resistance in Thailand: A Longitudinal Study

**DOI:** 10.1093/infdis/jix371

**Published:** 2017-08-03

**Authors:** Ricardo Ataíde, Rosanna Powell, Kerryn Moore, Alistair McLean, Aung Pyae Phyo, Shalini Nair, Marina White, Tim J Anderson, James G Beeson, Julie A Simpson, Francois Nosten, Freya J I Fowkes

**Affiliations:** 1 Disease Elimination Program, Burnet Institute,; 2 Centre for Epidemiology and Biostatistics, Melbourne School of Population and Global Health, University of Melbourne, and; 3 Department of Epidemiology and Preventive Medicine, Department of Infectious Diseases, Monash University, Melbourne,Australia;; 4 Shoklo Malaria Research Unit, Mahidol-Oxford Tropical Medicine Research Unit, Faculty of Tropical Medicine, Mahidol University, Mae Sot,Thailand;; 5 Texas Biomedical Research Institute, San Antonio;; 6 Centre for Tropical Medicine and Global Health, Nuffield Department of Medicine Research, University of Oxford, United Kingdom

**Keywords:** *Plasmodium falciparum*, malaria, artemisinin, drug resistance, immunity, antibodies

## Abstract

**Background:**

Reductions in malaria transmission decrease naturally acquired immunity, which may influence the emergence of *Plasmodium falciparum* artemisinin-resistant phenotypes and genotypes over time.

**Methods:**

Antibodies specific for *P. falciparum* antigens were determined in uncomplicated hyperparasitemic malaria patients over a 10-year period of declining malaria transmission and emerging artemisinin resistance in northwestern Thailand. We investigated the association between antibody levels and both parasite clearance time (PCt_½_) and artemisinin resistance–associated *kelch13* genotypes over time.

**Results:**

Immunity to *P. falciparum* declined prior to 2004, preceding the emergence of artemisinin resistance-associated genotypes and phenotypes (maximum mean change in antibody level per year: anti-MSP1_42_ = −0.17; 95% confidence interval [CI] = −.31 to −.04; *P* = .01). In this period of declining immunity, and in the absence of *kelch13* mutations, PCt_½_ increased. Between 2007 and 2011, levels of antibodies fluctuated, and higher antibody levels were associated with faster PCt_½_ (maximum yearly change in PCt_½_, in hours: EBA140_rII_ = −0.39; 95% CI = −.61 to −.17; *P* < .001).

**Conclusions:**

Understanding the impact of changing transmission and immunity on the emergence of artemisinin resistance is important particularly as increased malaria control and elimination activities may enhance immunological conditions for the expansion of artemisinin-resistant *P. falciparum*.

## Summary

A 10-year-long evaluation of immunity to *Plasmodium falciparum* and emergence of artemisinin resistance showed that immunity declined sharply in the years preceding the emergence of parasites with artemisinin-resistant phenotypes and genotypes and was associated with faster parasite clearance times.

Artemisinin combination therapies (ACT) are recommended by the World Health Organization (WHO) as the first-line treatment for *Plasmodium falciparum* malaria [1]. Artemisinin resistance, defined by the presence of microscopically detectable *P. falciparum* parasites on the 3rd day of artemisinin treatment, or prolonged parasite clearance half-life (PCt_½_) [2], was independently reported in western Cambodia in 2009 [3–7], followed by western Thailand [8], southern Myanmar [9, 10], and southern Vietnam [11]. In 2014 mutations in the “propeller” region of the *P. falciparum* Kelch protein encoded on chromosome 13 (*kelch13*) were identified as a genetic marker of artemisinin resistance [12–14]. The presence of *kelch13* mutations, together with a slow-clearing phenotype (PCt_½_ ≥ 5 hours), was used to confirm that artemisinin resistance is now firmly established in the Greater Mekong Subregion—western Cambodia, Thailand, eastern Myanmar, and southern Vietnam—and is emerging in northern Cambodia and southern Laos [12]. To date, no artemisinin resistance–associated mutations have been reported in Africa, despite the wide distribution of nonsynonymous mutations present in the *kelch13* gene [15].

In the Greater Mekong Subregion, artemisinin resistance–associated mutations and phenotypes are expanding as well as emerging independently [16]. This emergence and expansion will be influenced by many factors such as transmission, antimalarial treatment policies, public health interventions, the parasite population, and factors of the individual host harboring the infection. Naturally acquired antibody-mediated immunity to malaria, which develops after repeated exposure to *P. falciparum* [17], targets blood-stage parasites (merozoites and infected erythrocytes), lowering parasitemia [18], and sporozoite and gametocyte stages, reducing transmission between mosquitoes and humans [19–21]. In a large, multinational study of artemisinin resistance across 11 study sites in Southeast Asia with varying levels of *P. falciparum* transmission and naturally acquired immunity, we demonstrated that immunity is an important predictor of the slow-clearing phenotype, with higher levels of immunity associated with faster PCt_½_ [22]. Furthermore, we demonstrated that *kelch13* mutant parasites are emerging in areas with the lowest levels of blood-stage and transmission-blocking immunity ([22] and F. J. I. Fowkes, unpublished data). This suggests that immunity plays an important role in the emergence of resistant mutant parasites; populations with low levels of blood-stage immunity would be less effective at spontaneously eliminating mutant parasites and would more effectively transmit resistant parasites due to low levels of transmission-blocking immunity. The emergence of resistance where immunity and transmission is lowest is a major concern, particularly because many regions are transitioning to low malaria transmission due to intensified control and elimination efforts.

Over the past decade, increased malaria control efforts and the introduction of ACTs have led to substantial reductions in malaria transmission, morbidity, and mortality [23]. Reductions in malaria transmission can lead to a decline in naturally acquired immunity at the individual and population level [24]. We hypothesized that declining immunity over time resulting from a decline in malaria transmission would lead to increases in PCt_½_ after artemisinin treatment over the same interval. We tested this hypothesis at the Thai–Myanmar border, where there has been significant decline in malaria transmission (*P. falciparum* prevalence among 5-year-olds admitted to health clinics decreased by >80% between 2001 and 2010 [25, 26]) and artemisinin resistance emerged during the same time period (median PCt_½_ increasing from 2.6 hours [95% confidence interval {CI} = 2.5–2.7] in 2001 to 3.7 hours [95% CI = 3.6–3.8] in 2010 [8] to 7.2 hours [95% CI = 6.3–7.4] in 2014 [27]). In this study, we aimed to understand the associations between temporal changes in antibodies specific for *P. falciparum* in this population and the emergence of artemisinin resistance. Additionally, we aimed to quantify these changes with regards to the emergence of artemisinin-resistant phenotypes and genotypes over a 10-year period on the northwestern border of Thailand.

## METHODS

### Participants and Samples

Between 2001 and 2011, dried blood spots and plasma samples were obtained from 1732 and 896 hyperparasitemic falciparum malaria patients, respectively, who attended 4 malaria clinics (Mawkertai, Maela, Mae Khon Ken, Wang Pha) run by the Shoklo Malaria Research Unit (SMRU) along the northwestern border of Thailand. Clinical and data collection procedures have been described previously [8]. Briefly, patients included in this analysis were those diagnosed with uncomplicated hyperparasitemic falciparum malaria (>4% parasitemia and no signs of severe malaria) who were administered treatment with a 7-day regimen of oral artesunate (4 mg/kg initially, then 2 mg/kg once daily for 7 days), usually combined with mefloquine (25 mg/kg in 2 divided doses) or doxycycline (4 mg/kg per day for 7 days) or clindamycin (5 mg/kg 3 times daily for 7 days) if mefloquine was contraindicated. *Plasmodium falciparum* infection was confirmed by microscopy using both thick and thin peripheral blood smears stained with Giemsa. Patients were hospitalized and monitored every 6 hours by blood smear until smears were parasite-negative in order to calculate parasite clearance half-life after artemisinin treatment [2]. Admission blood spots were used to extract parasite DNA for *kelch13* genotyping, which was performed at the Texas Biomedical Research Institute in San Antonio, Texas (detailed in [8] and [27]). For each study site and year, all available dried blood spots were selected for antibody determination, except for the site of Wang Pha, which had a high number of blood spots available; thus a maximum of 130 blood spots from Wang Pha were randomly selected for this study. Dried blood spots were collected from patients and stored at −20°C in individual sealed plastic bags containing desiccant beads. Samples were then sealed in 2 outer plastic bags to ensure they were kept dry. Plasma was stored at −80°C until shipped to Melbourne, Australia. The collection and use of samples for this study were approved by the ethics review boards of the Faculty of Tropical Medicine, Mahidol University, Thailand; the Oxford Tropical Research Ethics Committee (no. 28-09); and the Alfred Hospital, Melbourne, Australia (no. 485-12).

### Measurement of Anti–*Plasmodium falciparum* Antibodies

Total immunoglobulin G (IgG) was determined toward the *P. falciparum* 3D7 merozoite antigens MSP1_42_ (amino acids 1362–1720), AMA-1 (whole ectodomain, amino acids 25–545), and MSP-2 (whole ectodomain, amino acids 19–249) (expressed in *Escherichia. coli*, his-tagged) and EBA140_RII_ (whole region; expressed in *Pichia pastoris*, also his-tagged). These antigens are thought to play a role in erythrocyte invasion and have been assessed as biomarkers of immunity to malaria [28]. Briefly, plates were coated with antigen (0.5 μg/mL, 50 μL per well), incubated overnight at 4°C, then blocked for 2 hours at 37°C. Samples were incubated for 2 hours at room temperature (see dilutions below). Secondary anti-human IgG labeled with horseradish reroxidase was then added at a dilution of 1/2000 in PBS 0.05% Tween-20 and 0.01% casein and incubated for 1 hour at room temperature. Plates were washed 3 times, and substrate was added. The reaction was stopped using 1% sodium dodecyl sulfate, and samples were read at 405 nm.

### Dried Blood Spot Samples

Sera was eluted off dried blood spots by punching the filter paper and placing a single 3-mm disk in 150 µL of phosphate-buffered saline with Tween (0.05%) and Azide (0.02%) overnight in a low-affinity 96-well plate on an automated plate shaker at 4°C. The eluted antibodies were used to measure the level of anti–*P. falciparum* antibodies through enzyme-linked immunosorbent assay. Eluted sera were added to the plates with 0.01% casein (roughly a 1/200 dilution from original spotted blood).

Suboptimal storage of filter papers can lead to poor recovery of antibodies from filter paper spots [17]. Pilot studies were performed using 90 samples from each year from 2001 to 2011 to determine whether the length of storage of dried blood spots would influence antibody levels. Few samples from before 2007 showed high antibody reactivity (only 1 sample had an optical density [OD] > 0.2; OD = 0.78), so only samples from 2007 to 2011 were selected for antibody determination by dried blood spot (n = 1143). Antibodies to MSP1_42_ and AMA1 were determined in 1143 samples. Antibody levels to EBA140_RII_ and MSP2 were determined in 1068 samples due to insufficient sample volume in 74 samples.

### Plasma Samples

For plasma samples, dilutions used were 1/1000 for antigens AMA-1 and EBA140_RII_, 1/500 for MSP1-42, and 1/250 for MSP-2. Plasma samples obtained from Melbourne (malaria-unexposed) individuals were used as negative controls.

### Statistical Analyses

#### Antibody Levels Over Time

Univariate linear regression models were fitted to determine the association between time (date of admission) and total plasma IgG for each antigen. Plasma samples were collected between July 2001 and December 2011; however, there was a paucity of samples available in 2005 (n = 4) and 2006 (n = 0), so these 2 years were excluded from the analysis. Lowess curve fitting analysis revealed 2 distinct segments in the associations between time and immunity, so we subsequently fit models with two segments (July 2001 to December 2004, and November 2007 to December 2011). After univariate regression models were specified, we incorporated possible confounder variables (study site and age) to account for changes in the population over time. All models met the assumption of normally distributed residuals.

#### Antibody Levels and Parasite Clearance Half-Life

Parasite clearance half-life was derived using the parasite clearance estimator [2]. Of 1732 samples available for this study, PCt_½_ was not available for 311 (18%) patients who did not have the required frequency of parasite data sampled; all remaining patients had sufficient parasite count data available for calculation of PCt_½_. Because the age of the dried blood spots influenced antibody elution (Supplementary Figure 1) [29], antibody data were ranked within each year and classified as high or low based on falling above or below the calendar year–specific median ranked value, respectively. The association between antibody levels (high vs low) with PCt_½_ was assessed using multivariable linear regression with adjustment for potential confounders: year of admission, study site, and age of patient. An interaction between antibody response with year and study site was examined to determine whether the magnitude of difference in PCt_½_ according to immunity varied according to year or site of data collection. Interactions with *kelch13* genotype were also assessed where genotype data were available. Mutations in the *P. falciparum kelch13* gene above amino acid position 440, present in >5 individuals and with a median PCt_½_ ≥ 5 hours were defined as a *kelch13* mutant associated with resistance. Models with and without interaction terms were compared using the likelihood ratio test. One influential outlier was removed from the parasite clearance analysis (PCt_½_ = 23.7 h) because it changed coefficients by >10%. All models met the assumption of normally distributed residuals. All analyses were performed using STATA 13.1 (StataCorp, College Station, TX).

## RESULTS

### Study Area and Population

**Figure 1.**
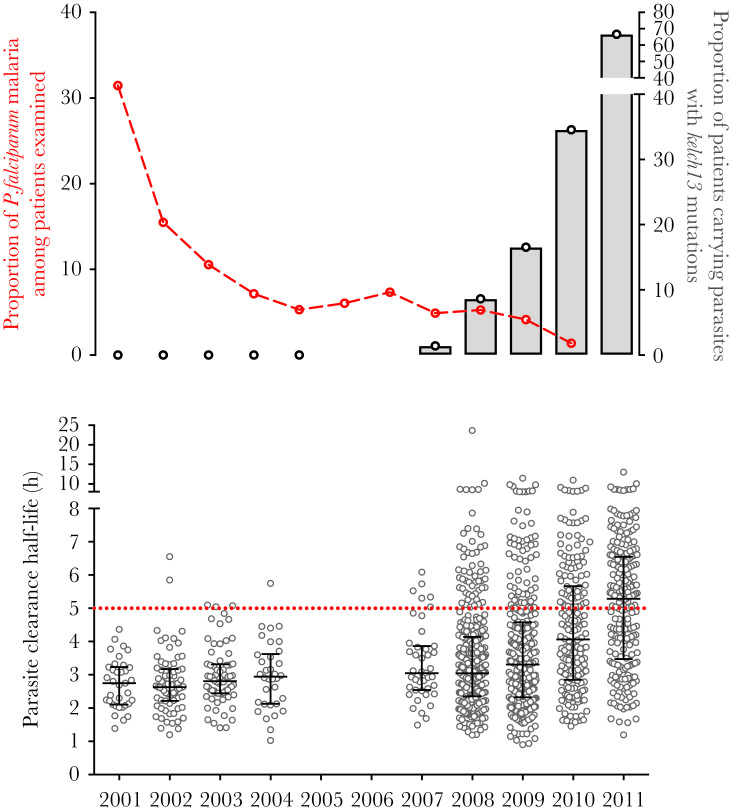
Changes in transmission intensity, proportion of *kelch13* mutations, and parasite clearance rates from 2001 to 2011. *Upper graph:* Proportion of *Plasmodium falciparum*–positive cases among children aged <5 years attending clinic within the same study area (left *y*-axis, data adapted from [25]); Proportion of patients genotyped with a *kelch13* mutation associated with parasite clearance half-life (PCt_1/2_) ≥ 5 hours (right *y*-axis). *Lower graph*: The PCt_1/2_ (medians and interquartile ranges) are plotted. Abbreviation: *P. falciparum*, *Plasmodium falciparum*.

Since 2001, malaria transmission has declined and artemisinin resistance has emerged at SMRU malaria clinics along the Thai–Myanmar border [8]. During 2001 and 2011, there was a decline in the proportion of falciparum malaria consultations among children aged <5 years admitted to study clinics, indicative of declining *P. falciparum* transmission in the study area (Figure 1). During this time period, *kelch13* mutations were retrospectively detected as early as 2003, and from 2007 onward, *kelch13* mutations associated with a slow-clearing phenotype (defined as mutations present in >5 individuals, at amino acid positions 441 and above, and with a median PCt_½_ ≥ 5 hrs), characteristic of artemisinin resistance parasites, increased in frequency (Figure 1). *Plasmodium falciparum* enrollment parasitemia at admission was similar over the study period among the patients in this cohort (eg, 2001: 290136 parasites/µL; 2011: 293339 parasites/µL) (Table 1). The majority of patients were males of working age, reflecting that the majority of malaria is associated with occupational exposure (Table 1).

**Table 1.** Characteristics of Included Uncomplicated Hyperparasitemic Falciparum Malaria Patients Admitted Between 2001 and 2011

Variable	**2001**	**2002**	**2003**	**2004**	**2007**	**2008**	**2009**	**2010**	**2011**
Plasma, no.	43	85	90	58	32	372	22	23	152
Dried blood spots, no.	0	0	0	0	44	362	315	193	229
Age, y, p50, p25–p75 (min, max)	13.0, 18.0–21.0 (5.0, 50.0)	9.0, 15.0–22.0 (1.0, 58.0)	8.0, 18.0–22.0 (0.0, 50.0)	8.0, 13.5–25.0 (1.0, 53.0)	5.0, 12.0–22.0 (2.0, 45.0)	6.0, 17.0–25.0 (0.0, 70.0)	6.0, 13.0–21.0 (0.0, 60.0)	6.0, 14.0–25.0 (0.0, 63.0)	8.0, 17.0–28.0 (0.0, 62.0)
Female, n/N (%)	12/35 (34.3)	23/72 (32.0)	26/68 (38.3)	9/34 (26.5)	16/44 (36.4)	128/362 (35.4)	121/315 (38.4)	76/193 (39.4)	65/228 (28.5)
Parasitemia, /µL, p50 (min, max)	290136 (66317, 1107164)	285991 (60288, 895528)	231418 (33158, 1033939)	281846 (44211, 1112188)	307092 (91437, 763146)	288126 (1520, 2011610)	272238 (32656, 1790554)	281344 (22608, 1276850)	293339 (45216, 2409008)
Gametocytes, n/N (%)	7/35 (20.0)	6/72 (8.3)	4/68 (5.9)	4/34 (11.8)	5/39 (11.4)	53/362 (14.6)	41/315 (13.0)	36/193 (18.7)	37/228 (16.2)
Patients treated with artesunate + mefloquine,^a^ n/N (%)	29/35 (82.90	65/72 (9.03)	59/68 (86.8)	28/34 (82.4)	27/44 (61.4)	292/362 (80.7)	249/315 (79.1)	157/193 (81.4)	144/228 (63.1)
Parasite clearance half-life, h, p50, p25–p75 (min, max)	2.10, 2.75–3.23 (1.39, 4.37)	2.21, 2.69–3.17 (1.20, 6.55)	2.45, 2.81–3.31 (1.41, 5.09)	2.13, 2.94–3.60 (1.03, 5.75)	2.54, 3.05–3.87 (1.49, 6.09)	2.36, 3.04–4.13 (1.19, 23.8)	2.32, 3.31–4.58 (0.90, 11.46)	2.86, 4.06-5.66 (1.45, 10.96)	3.50, 5.28–6.55 (1.20, 13.03)
No. of samples with K13 alleles genotyped^b^	7	18	15	11	26	303	298	138	112
K13 allele associated with artemisinin resistance/wild-type allele,^c^ n/N (%)	0/7 (0.0)	0/17 (0.0)	0/13 (0.0)	0/11 (0.0)	1/24 (4.2)	29/262 (11.1)	24/148 (16.2)	38/110 (34.6)	61/92 (66.3)

The number of matched plasma and dried blood spot samples were: 2007 (30); 2008 (241); 2009 (17); 2010 (22); 2011 (147).

^a^Remaining patients were given artesunate monotherapy, artesunate in combination with either doxycycline or clindamycine, or other combinations.

^b^ Number of patients with genotype data for *kelch13*.

^c^(n) is the number of individuals with *kelch13* mutations associated with artemisinin resistance (median PCt_1/2_ ≥ 5h and nonsynonymous mutations above amino-acid position 440); (N) is the number of individuals with wild-type *kelch13*.

### Changes in *Plasmodium falciparum* Transmission and Immunity Between 2001 and 2011

**Figure 2.**
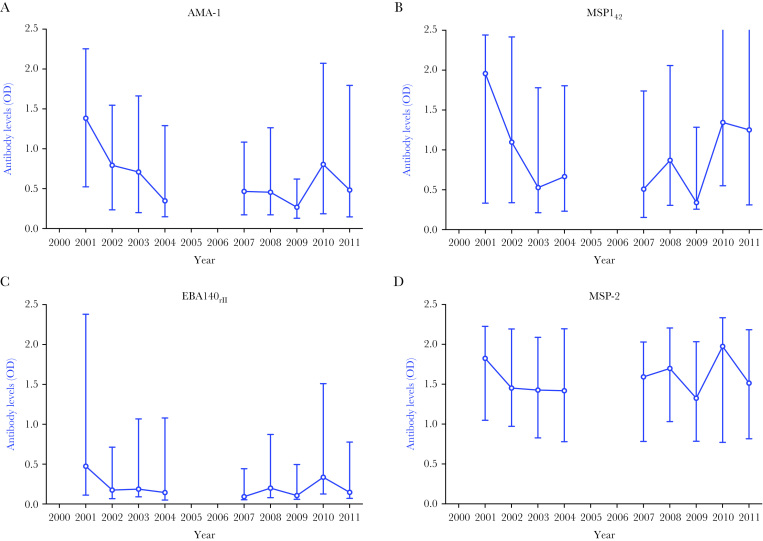
Levels of antibodies specific for *Plasmodium falciparum* in plasma samples, 2001 to 2011. *A–D,* Raw levels of plasma antibodies (optical density) obtained against the 4 antigens studied. Symbols and error bars represent medians and interquartile ranges, and lines connecting the medians of antibody values are there for illustrative purposes only. Estimates (95% confidence interval) of the magnitude of change of antibody levels against each antigen per year, after adjusting for study site, patient age, and enrollment parasitemia, are presented in Table 2. Abbreviation: OD, optical density.

To determine whether declining transmission was accompanied by declining immunity, antibody levels specific for the *P. falciparum* merozoite antigens AMA-1, MSP1_42_, MSP-2, and EBA140_RII_ were determined from all available plasma samples from 2001 to 2011. Antibody levels to each antigen were well correlated (Spearman’s rho range = 0.51–0.71). Between 2001 and 2004, observed antibody levels to *P. falciparum* antigens declined, which was followed by a period of fluctuation between 2007 and 2011 (Figure 2A–D). As levels of antibody varied according to clinic attended, age, and enrollment parasitemia, which were variable according to year of admission, a multivariable analysis was performed to examine changes in antibodies over time, adjusting for these confounding variables. Multivariable analyses showed that antibody responses to all antigens decreased between 2001 and 2004: AMA-1 (mean change in antibody level per year = −0.14; 95% CI = −.25 to −.04; *P* = .007), MSP1_42_ (mean change in antibody level per year = −0.17; 95% CI = −.31 to −.04; *P* = .01), EBA140_RII_ (mean change in antibody level per year = −0.08; 95% CI = −.17 to .02; *P* = .11), and MSP-2 (mean change in antibody level per year = −0.07; 95% CI = −.18 to .02; *P* = .11) (Table 2). However, there was no significant change in mean antibody levels specific for the 4 *P. falciparum* antigens between 2007 and 2011 (mean change per year for all antibodies between −0.04 [95% CI = −.08 to .01] and 0.04 [95% CI = −.02 to .10]; all *P* ≥ .10) (Table 2). These results show that antibodies specific for relatively conserved *P. falciparum* antigens declined between 2001 and 2004, coinciding with the large decrease in *P. falciparum* transmission in the population (Figure 1).

**Table 2.** Adjusted Estimated Mean Difference in Plasma Samples Immunity Levels According to Year of Admission, Study Site, Age of Individual, and Enrollment Parasitemia Before Treatment

Variable	**AMA-1**		**MSP142**		**MSP-2**		**EBA140rII**	
	**Coefficient** **(95% CI)**	***P*** value	**Coefficient** **(95% CI)**	***P*** value	**Coefficient** **(95% CI)**	***P*** value	**Coefficient** **(95% CI)**	***P*** value
**Year of admission**								
2001–2004 (per year)	−0.14 (−.25 to −.04)	.007	−0.17 (−.31 to −.04)	.01	−0.07 (−.17 to .02)	.11	−0.08 (−.18 to .02)	.11
2007–2011 (per year)	0.02 (−.02 to .07)	.34	0.04 (−.02 to .10)	.18	−0.04 (−.08 to .01)	.10	−0.02 (−.06 to .03)	.49
**Site**								
Mae Khon Ken	Reference		Reference		Reference		Reference	
Mawker Thai	0.02 (−.22 to .25)	.88	0.00 (−.30 to .29)	.99	−0.05 (−.25 to .15)	.62	−0.03 (−.20 to .25)	.82
Maela	0.02 (−.23 to .27)	.86	−0.15 (−.47 to .16)	.34	−0.11 (−.33 to .11)	.32	−0.05 (−.29 to .19)	.68
Wang Pha	−0.10 (−.32 to .12)	.39	−0.04 (−.31 to .24)	.79	−0.02 (−.21 to .17)	.81	0.07 (−.14 to .28)	.53
**Age (per 5 yrs**)	0.08 (.06 to .10)	<.0001	0.04 (.01 to .07)	.004	0.03 (.02 to .05)	<.0001	0.07 (.05 to .09)	<.0001
**Parasitemia (per 100000 parasites**)	−0.04 (−.06 to −.01)	.002	−0.01 (−.04 to .02)	.46	−0.03 (−.05 to −.01)	.002	−0.04 (−.07 to −.02)	<.0001

Average changes in immunity levels by study period from multivariable regression models, including study site, patient age (average change per 5 years), and enrolment parasitemia (average change per 100000 parasites). All models were adjusted for study site. Time was modeled by including year of admission as a continuous variable with splines to examine differences in the 2 time periods.

### Changes in Immunity to *Plasmodium falciparum* and the Emergence of Artemisinin Resistance Between 2001 and 2011

**Figure 3.**
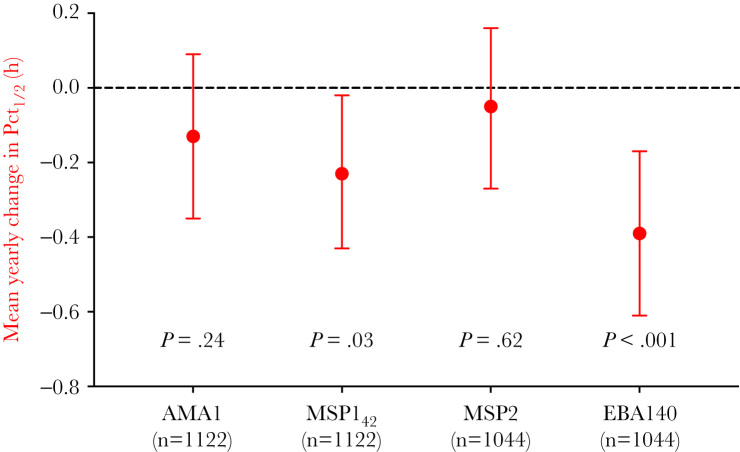
Mean yearly change in parasite clearance half-lives (PCt_1/2_) between seropositive and seronegative patients between 2007 and 2011. Data obtained from multivariable regression models of PCt_1/2_, including antibody variable, admission year, study site, and patient age (continuous). Data are represented as mean estimate and 95% confidence intervals. Dotted line indicates where no change in PCt_1/2_ would occur. There were 1122 AMA1 and MSP1_42_ samples and 1044 MSP2 and EBA140_rII_ samples available for this analysis. Abbreviation: PCt_1/2_, parasite clearance half-life.

The large decrease in *P. falciparum* transmission and *P. falciparum*–specific antibody levels between 2001 and 2004 preceded the expansion of *P. falciparum kelch13* mutants and slow-clearing parasites (Figure 1). Between 2007 and 2011, PCt_½_ increased from a median of 3.04 hours (95% CI = 1.85–5.52) in 2007 to 5.28 hours (95% CI = 2.14–7.94) in 2011, coinciding with the increasing prevalence of *kelch13* mutations (Figure 1). High antibody levels to MSP1_42_ and EBA140_rII_ were associated with a moderately shorter PCt_½_ (estimated mean difference in PCt_½_: MSP1_42_ = −0.23 [95% CI = −.43 to −.02] hours, *P* = .03; EBA140_rII_ = −.39 [95% CI = −.61 to −.17] hours, *P* < .001), whereas high antibody responses to AMA-1 and MSP-2 were still associated with a decrease in PCt_½_, but with smaller magnitudes of effect (Figure 3). There was no evidence that the association between antibody responses and PCt_½_ was modified by year of admission (all *P* values for interaction >.13), indicating that the magnitude of difference in parasite clearance time in those with high and low responses did not vary between 2007 and 2011. Furthermore, in a subset of 557 patients, where both antibody and *kelch13* genotype data were available, there was no evidence of an interaction between antibody responses and presence or absence of *kelch13* mutations associated with artemisinin resistance (all *P* > .34), indicating that the magnitude of difference in PCt_½_ between high and low responses did not vary according to the presence of *kelch13* mutations.

## DISCUSSION

In this longitudinal study, we demonstrate important associations between *P. falciparum* transmission, immunity, and the emergence of artemisinin-resistant falciparum malaria over a 10-year period in northwest Thailand. We found that immunity to *P. falciparum* predominantly declined prior to the emergence and expansion of artemisinin resistance–associated genotypes and phenotypes from 2003. Between 2007 and 2011, levels of antibodies specific for *P. falciparum* did not follow any particular trend, and high antibody levels were associated with moderately faster parasite clearance rates.

In this region of northwestern Thailand, *P. falciparum* transmission and immunity declined during 2000 and 2004 prior to or during the first stages of emerging *kelch13* resistance–associated mutations. A number of factors may have contributed to this observed temporal relationship. First, artemisinin-based therapy was introduced in this region of Thailand in 1995 [30]. The introduction of these highly efficacious therapies contributed significantly to the large reductions observed in *P. falciparum* transmission and associated reductions in immunity between 2000 and 2004 and provided the drug pressure required for the selection of mutations that confer artemisinin resistance. Second, the drop in transmission led to a decrease in the proportion of infections containing multiple *P. falciparum* genotypes (63% in 2001 to 14% in 2010) in this study population [25]. This may reduce within-host competition between resistant and sensitive genotypes. Furthermore, reductions in the number of infections containing multiple genotypes results in higher rates of parasite inbreeding [31, 32], which may increase the rate of spread of drug resistance when multiple loci are involved. Therefore drug resistance mutations are more likely to emerge, establish, and spread in low transmission areas. Last, we hypothesize that declining immunity may be an important factor for the survival and expansion of parasites carrying resistance-associated *kelch13* mutations with a lower fitness than wild-type parasites [33]. Immune individuals are more likely to respond well to antimalarial treatment and require shorter treatment regimens (even when drug-resistant parasites are present) (reviewed in [34]). This effectively turns immune individuals into refuges for drug-sensitive parasites, halting the spread of resistance [35]. Although in this study it is hard to dissect out the relative contributions of drug pressure and changing transmission and immunity on temporal cause-and-effect mechanisms, results are in concordance with our previous multinational, cross-sectional study that showed that the highest frequencies of *kelch13* mutations are found in areas of lowest immunity, even in areas where artemisinin therapy was introduced as first-line policy at similar times [22]. Temporal relationships are most important to understand in areas where artemisinin resistance is yet to emerge, such as sub-Saharan Africa, which harbors the greatest burden of malaria and where several efforts are in place to reduce transmission. Artemisinin derivatives were significantly scaled up in Africa in 2007, which has subsequently seen large reductions in transmission [36] and naturally acquired immunity [37–39] over the same time period. Although *kelch13* resistance–associated mutations are yet to emerge in Africa [15], the changing epidemiology of malaria and wide-scale use of artemisinin-based therapies in the region highlight the need for close monitoring of resistance to artemisinin.

Between 2007 and 2011 when immunity was relatively low, we found that individuals with high levels of antibodies had faster PCt_½_ compared with those with low antibody levels, and a subanalysis showed that this effect was similar in patients with wild-type and *kelch13* mutant parasites. The magnitude of effect varied according to antigen, with the largest differences of −0.23 and −0.39 hours observed for MSP142 and EBA140_rII_, respectively. These magnitudes of effect are in concordance with results from our previous multinational, cross-sectional study, which showed that *P. falciparum* antibody responses were associated with a reduction of PCt_½_ of −0.52 to −0.12 hours, depending on antigen [22]. However, both of these studies included patients with high parasitemias (>4% infected erythrocytes in the longitudinal study and >10000 parasites/µL in the multinational study), so the generalizability of these magnitudes of effect of immunity on PCt_½_ observed in patients whose immune responses are unable to control parasite multiplication to patients with lower parasitemias is yet to be determined. The magnitude of effect did not vary according to the frequency of *kelch13* resistance–associated mutations, which increased from 4.2% in 2007 to 66.3% in 2011 in our study sample. We have previously shown that the effect of immunity on PCt_½_ is similar in areas with varying frequencies of *kelch13* mutations [22]. The magnitude of effect of *P. falciparum* antibodies on PCt_½_ did not vary with year between 2007 and 2011, potentially because antibody levels in hyperparasitemic patients were relatively constant during this time. Additionally, the categorization of antibody levels determined in dried blood spot samples as high or low within each year, to overcome measurement bias with improved antibody elution over time, may have biased magnitudes of effect in the association between *P. falciparum* antibodies and PCt_½_ as well as assessments of effect modification of this association with time and *kelch13* genotypes. The comparisons of high versus low antibody levels using a median cutoff may also result in nondifferential misclassification of clinically relevant antibody thresholds (because immunogenicity varies according to antigen) in the high/low categories and bias findings towards the null. For example, AMA-1 is known to be highly immunogenic [40], and comparisons of the high versus low categories may actually be a comparison of high versus very high groups, which may be equally associated with PCt_½_. Despite this potential misclassification, we were able to show similar associations and magnitudes of effect between antibodies specific for certain *P. falciparum* antigens and PCt_½_ compared with our previous multinational study using plasma [22]. The consistency of these findings validates the potential use of dried blood spots for serosurveillance studies of artemisinin therapeutic efficacy and tracking changing malaria transmission in the population.

With the recent release of the Strategy for Malaria Elimination in the Greater Mekong Subregion (2015–2030) [41] and its goal to control and eliminate malaria from this region, it is important to understand the factors that may contribute to the emergence of artemisinin resistance in a landscape of changing transmission. Furthermore understanding how changing immunity may affect parameters in the WHO definition of artemisinin resistance is important to inform artemisinin resistance monitoring and surveillance efforts. Our study shows important ecological temporal relationships between transmission, levels of immunity, and the emergence of artemisinin-resistant phenotypes and genotypes. Understanding the impact of changing transmission and immunity on the emergence of resistant parasites is important particularly because increased malaria control and elimination activities may enhance conditions for the expansion of artemisinin-resistant *P. falciparum*.

## Supplementary Data

Supplementary materials are available at *The Journal of Infectious Diseases* online. Consisting of data provided by the authors to benefit the reader, the posted materials are not copyedited and are the sole responsibility of the authors, so questions or comments should be addressed to the corresponding author.

## Supplementary Material

Fig S1

Supplementary Figure Legend

## Notes

***Acknowledgements.*** We would like to thank Chris Drakeley and Patrick Corran for technical advice on extracting antibodies from dried blood spots.

***Financial support.*** This work was supported by the National Health and Medical Research Council of Australia (NHMRC; training fellowship no. GNT1060785 to F. J. I. F.; senior research fellowship no. 1104975 to J. A. S. and no. 1077636 to J. G. B.; project grant no. 1046575 to F. J. I. F., J. A. S., and F. N.), Australian Research Council (Future Fellowship number FT130101122 to F. J. I. F.), Ramaciotti Establishment grant (to F. J. I. F.). Funding from the Victorian State Government Operational Infrastructure Support scheme and the NHMRC Independent Research Institutes Infrastructure Support Scheme supported the Burnet Institute. The clinical study was done as part of the Wellcome Trust–Mahidol University–Oxford Tropical Medicine Research Program (grant B9RTOZ2) supported by the Wellcome Trust of Great Britain. Work in San Antonio was funded by the National Institutes for Health (R37 AI048071 to T. A.) and the Bill and Melinda Gates Foundation (grant no. OPP1040463) and was conducted in facilities constructed with support from a Research Facilities Improvement Program grant (grant no. C06 RR013556) from the National Center for Research Resources. The funders had no role in study design, data collection and analysis, decision to publish, or preparation of the manuscript.

***Potential conflicts of interest.*** All authors: No reported conflicts of interest. All authors have submitted the ICMJE Form for Disclosure of Potential Conflicts of Interest. Conflicts that the editors consider relevant to the content of the manuscript have been disclosed.
