# Supplementary figures and images for: Declining Transmission and Immunity to Malaria and Emerging Artemisinin Resistance in Thailand: A Longitudinal Study
Source: J Infect Dis. 2017 Aug 3;216(6):723–31. doi: 10.1093/infdis/jix371 (PMC5853569; doi:10.1093/infdis/jix371)

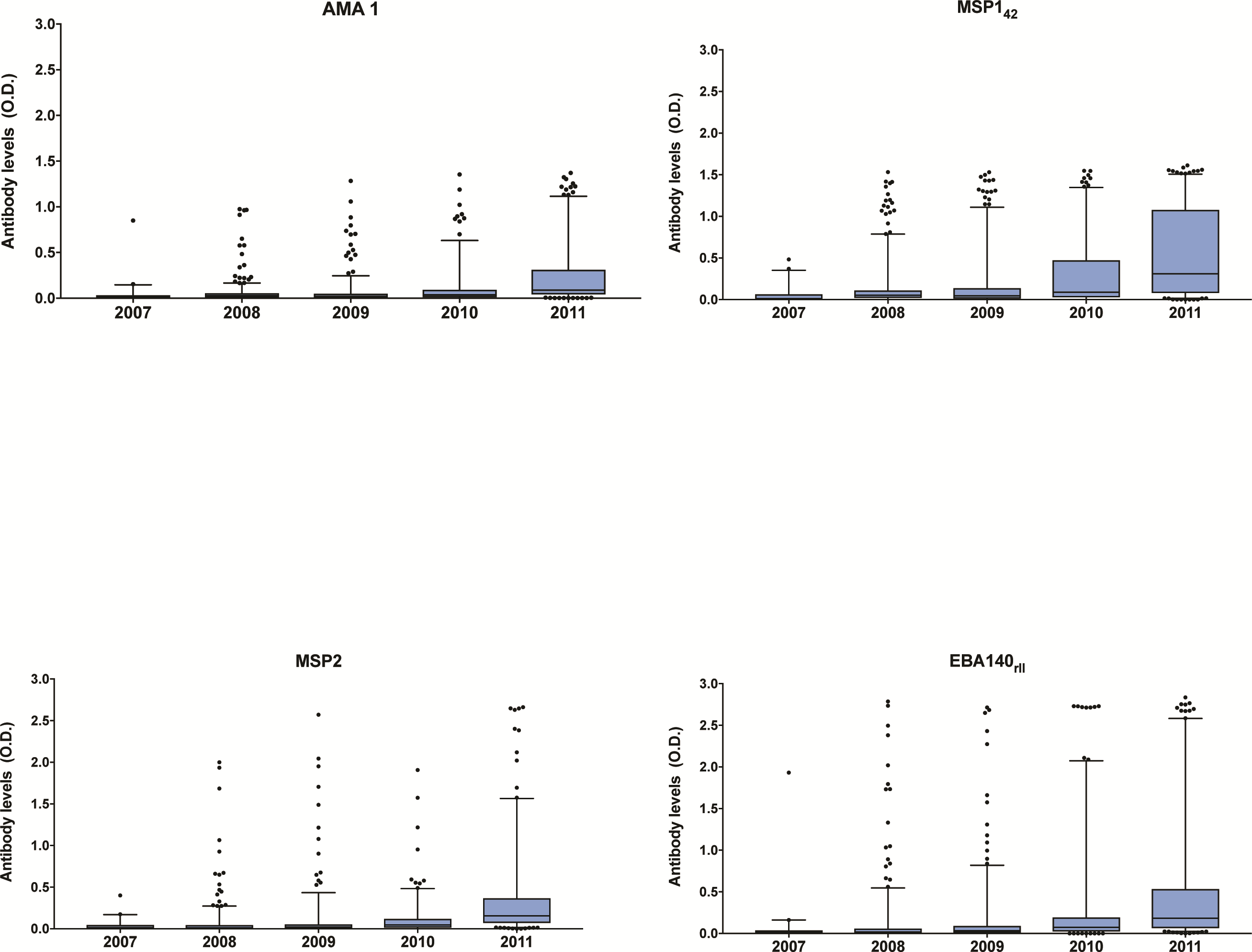

Supplement: Fig S1 [file jix371_suppl_figs1.png]
