# Supplementary material for: Declining Transmission and Immunity to Malaria and Emerging Artemisinin Resistance in Thailand: A Longitudinal Study
Source: J Infect Dis. 2017 Aug 3;216(6):723–31. doi: 10.1093/infdis/jix371 (PMC5853569; doi:10.1093/infdis/jix371)
Supplement: Supplementary Figure Legend [file jix371_suppl_supplementary-figure-legend.docx]

**SUPPLEMENTARY FIGURE LEGEND:**

**Supplementary figure 1 - Antibody levels eluted from dried blood spot samples from 2007 to 2011.** For all antigens, analyses of variance according to Kruskal-Wallis test, P < 0.001. Only 0.1% of dried blood spot samples (1/560 for 2 antigens) had detectable *P. falciparum* antibodies in eluates from samples collected prior to 2007 compared to >75% of plasma samples. Analyses of individuals with both antibody measurements showed an increase in the correlation between antibodies determined from dried blood spots and plasma in later years, e.g. AMA1 2007, spearmans rho = 0.278; 2008, rho = 0.223; 2009, rho = 0.332; 2010, rho = 0.543; 2011, rho = 0.826. We therefore concluded that antibody levels recovered from eluted dried blood spot samples were improved in samples collected more recently, a finding consistent with antibody degradation in dried blood spots with time, as reported in previous studies [29]
